# RE-EVALUATING LOCUS OF CONTROL AMONG OLDER KAREN REFUGEES IN LONG-TERM RESETTLEMENT

**DOI:** 10.1093/geroni/igae098.0767

**Published:** 2024-12-31

**Authors:** Noelle Fields, Katherine Kitchens, Diane Mitschke

**Affiliations:** The University of Texas at Arlington, Arlington, Texas, United States; The University of Texas Arlington, Arlington, Texas, United States; The University of Texas Arlington, Arlington, Texas, United States

## Abstract

Recent literature has increasingly applied the concept of locus of control (LOC), a psychological measure for understanding individuals’ beliefs about controlling life events, to forcibly displaced populations, including older refugees whose experiences and coping mechanisms may be distinctly influenced by their age and cultural background. The cross-cultural validity of this construct, particularly among collective cultures experiencing long-term resettlement in the Global North, warrants further examination. This study, utilizing interpretative phenomenological analysis within the theoretical framework of homeostatic socio-cognitive systems theory, specifically explored the lived experiences of forced displacement and long-term resettlement among ten older Karen refugees from Burma resettled in the southwestern United States for an average of over 12 years. Aimed at capturing deep narrative insights, the research sought to extend the discourse on LOC’s cross-cultural construct validity, particularly within the context of older refugees adapting to life in a Western context. The findings underscored five emergent themes: Collective Orientation over Individual Agency, Collective Cultural and Communal Living, Interdependence and Communal Support, Shared Control within Social Networks, and the Importance of Relationships. These themes reveal a significant divergence from the individualistic assumptions underlying the LOC construct, highlighting the prominence of communal values, shared decision-making, and the critical role of relational dynamics within the collective cultures of older refugees. This study underscores the need for culturally sensitive research methodologies and intervention strategies that honor the unique lived experiences of older refugees from collective cultures, advocating for a reevaluation of psychological constructs like LOC in cross-cultural contexts.

